# Sentinel Lymph Node Detection by 3D Freehand Single-Photon Emission Computed Tomography in Early Stage Breast Cancer

**DOI:** 10.4274/mirt.81894

**Published:** 2016-06-06

**Authors:** Salih Sinan Gültekin, Ahmet Oğuz Hasdemir, Serhat Tokgöz, Gülay Özgehan, Hakan Güzel, Cüneyt Yücesoy, Emine Öztürk, Ata Türker Arıkök

**Affiliations:** 1 Dışkapı Yıldırım Beyazıt Training and Research Hospital, Clinic of Nuclear Medicine, Ankara, Turkey; 2 Dışkapı Yıldırım Beyazıt Training and Research Hospital, Clinic of Surgery, Ankara, Turkey; 3 Dışkapı Yıldırım Beyazıt Training and Research Hospital, Clinic of Radiology, Ankara, Turkey; 4 Dışkapı Yıldırım Beyazıt Training and Research Hospital, Clinic of Pathology, Ankara, Turkey; 5 Hacettepe University Kastamonu Faculty of Medicine, Department of Nuclear Medicine, Ankara, Turkey

**Keywords:** Breast cancer, Sentinel lymph node biopsy, radionuclide tomography, single photon emission computed tomography, computer assisted three dimensional imaging, image guided surgery

## Abstract

We herein present our first experience obtained by 3D freehand single-photon emission computed tomography (SPECT) (F-SPECT) guidance for sentinel lymph node detection (SLND) in two patients with early stage breast cancer. F-SPECT guidance was carried out using one-day protocol in one case and by the two-day protocol in the other one. SLND was performed successfully in both patients. Histopathologic evaluation showed that the excised nodes were tumor negative. Thus, patients underwent breast-conserving surgery alone.

## Abstract (tr)

Bu yazıda erken evre meme kanserli iki hastada sentinel lenf nodu tespiti (SLNT) için bilgisayarlı tek foton emisyonlu tomografisi (SPECT) 3D freehand SPECT (F-SPECT) rehberliği yoluyla edinilen ilk deneyimimizi sunuyoruz. F-SPECT rehberliği bu olguların birisinde tek gün protokolü diğerinde iki gün protokolü kullanılarak gerçekleştirildi. SLNT her iki hastada başarı ile gerçekleştirildi. Histopatolojik inceleme çıkarılan nodların tümör negatif olduğunu gösterdi. Hastalar bu sayede sadece meme koruyucu cerrahi geçirdiler.

## INTRODUCTION

Breast surgery has evolved from radical mastectomy to minimally invasive procedures in time. Axillary node status is the main prognostic factor in the management of breast cancer patients. Nodal axillary involvement is shown in 10-30% of the cases with a T1 tumor, and in 45% of T2 tumors (1). Routine axillary lymph node dissection may cause additional risks such as lymphedema, persistent pain, and sensory impairment. Sentinel lymph node detection (SLND) allows information about the first nodal basin of lymphatic flow, and to decide whether axillary dissection by a minimally invasive approach is required or not. Currently, SLND is more favorable than routine axillary node dissection in accordance with recent expert views (1,2,3). With the introduction of SLND concept, radio-guided surgery has become a popular technique in breast cancer patients (1,2,3). Preoperative lymphoscintigraphy and intraoperative detection methods (hand-held gamma probe or blue dye) with one-day or two-day protocols (4) have become widely used methods despite some limitations. Single photon emission computed tomography (SPECT)/computed tomography (CT) hybrid imaging could yield better results in certain cases (5). Today, research&development studies have recently focused on new imaging probes that contain an intraoperative counting probe integrated with a small-field-of-view gamma camera (5,6,7,8,9). In this paper, we present our first experiences for SLND in two early-stage breast cancer patients by using a three-dimensional (3D) freehand SPECT (F-SPECT) device (declipse SPECT®, SurgicEye, Munich, Germany).

## CASE REPORTS

### Case 1

**Figure 1:**
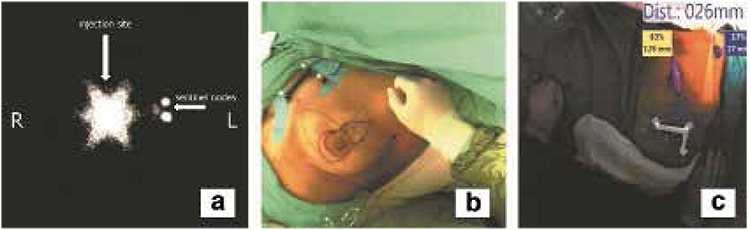
The lymphoscintigraphic image of the left breast on the left anterior oblique projection 30 min after radiotracer injection shows tracer accumulation in the injection site (long white arrow) and two adjacent axillary sentinel lymph nodes (short white arrow), b) The view of the operation theatre. Skin mark belonging to the sentinel lymph node is seen in the left axillary region, c) In the operating room, live video image shows two distinct radiotracer uptake foci in the injection site (rate: 83%, depth: 128 mm) and axillary region (rate: 17%, depth: 27 mm)

**Figure 2:**
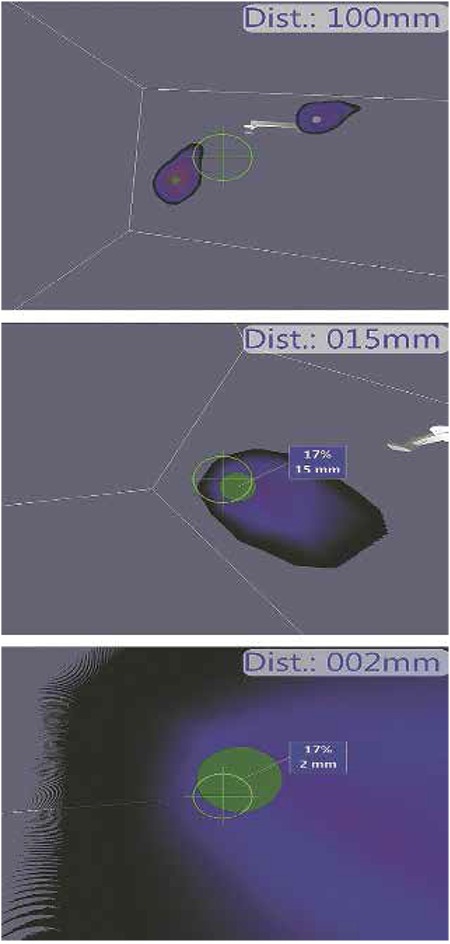
Three-dimensional navigation. The figure depicts the distance from the tip of the gamma navigation probe to the hot spot (respectively, top-down; 100 mm, 15 mm, 2 mm). Target sign defines the probe direction and optimal access path to the radioactive area

**Figure 3:**
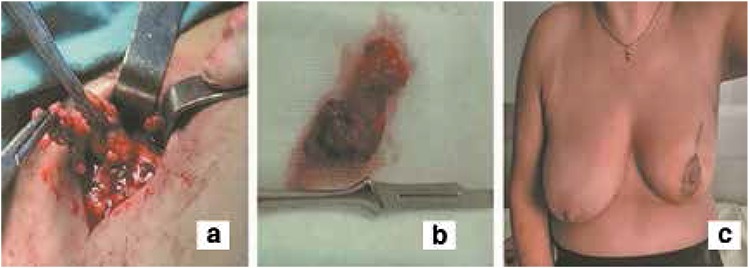
The figure shows axillary lymph node dissection during surgery, b) Surgical specimen belonging to the excised lymph nodes due to high radiotracer uptake (165 cps/s), c) The postoperative status of the left breast at tenth day after the surgery

A 54-year-old postmenopausal woman was evaluated for a left-sided breast mass. A 14x11 mm mass located in the upper-middle part of the left breast with a breast imaging-reporting and data system (BI-RADS) 5 score was determined at breast ultrasound and mammography. The patient’s cancer was staged as IA (T1N0M0). Preoperative lymphoscintigraphic images were obtained using a large field of view gamma camera (ECAM, Siemens, Illinois, USA) on anterior and oblique positions at 5, 30 and 60 min. following the periareolar-subdermal injection of 99mTc-labeled nano-colloid in a dose of 74 MBq/0.2 mL to the left breast. In the scintigraphic image, the two focal uptakes in nodal basin (Figure 1a) were considered as sentinel lymph nodes (SLNs). These foci were marked on skin in the left axillary region (Figure 1b). The patient underwent 3D F-SPECT guided breast surgery with a two-day protocol. 24h after tracer injection, we scanned the breast and the axilla for a 2-4 min period in different directions (1 min each in the medial, dorsal and medial-dorsal directions) according to the procedure described by Wendler et al. (8). A sufficient count rate was determined by using a color code in green. The number and position of the sentinel nodes were observed on a computer screen and the completion of the surgical procedure was confirmed by repeat screenshots. 3D F-SPECT revealed two main radiotracer uptakes on the injection site (83%) and in the left level I axillary lymph nodes (17%) (Figure 1c). The lymphatic hot spot (mean count rate: 165 cps/s) was reached through a skin incision under the guidance of realtime 3D navigation definitions (Figure 2). After histological frozen section examination of the excised SLNs was reported as tumor negative, the procedure was completed with breast-conserving surgery with safe surgical margins (Figure 3). Final histopathology of the tumor revealed infiltrative ductal carcinoma.

### Case 2

A 40-year-old premenopausal woman had a 21x15 mm mass in the upper-outer quadrant of her right breast compatible with BI-RADS 4c score on breast ultrasound and mammography. The patient was classified as Stage IIA (T2N0M0) breast cancer according to the imaging studies. A tru-cut biopsy was reported as infiltrative ductal carcinoma. Preoperative lymphoscintigraphy and F-SPECT guided surgery was performed using same radiotracer, injection and imaging techniques with the one-day protocol. Scintigraphic examination showed high nodal uptake in the right axillary region and this area was marked on the skin. 4 hours after radiotracer injection, breast surgery was started with the guidance of 3D F-SPECT imaging. The real-time image from 3D F-SPECT detected meaningful radiotracer uptakes in two hot spots on the injection site and the right axilla. The hot spot which was located within the right level II axillary lymph nodes was accessed (mean count rate: 322 cps/s) and removed surgically. Frozen section evaluation revealed reactive lymph nodes that were tumor negative. The surgeon completed the operation with breast-conserving surgery with safe surgical margins.

## LITERATURE REVIEW AND DISCUSSION

Conventional nuclear medicine procedures have been reported to be useful for sentinel node detection (1,2). However, performance of the classical test for breast cancer showed a wide variation with a false-negative rate ranging from 0% to 29% as stated in a meta-analysis of 69 trials (10). The median false-negative rate was higher than 5% in all landmark studies with some inadequate results in special patient sub-groups (1,2,10,11). Therefore, researchers are trying to develop novel imaging tools and technology to improve these results. The introduction of portable gamma camera technology to clinical and experimental field enabled a rapid development. Mini gamma cameras, F-SPECT, and fluorescence imaging systems have been suggested for image-guided SLND. Mini gamma camera systems serve as a two-dimensional imaging modality. They were reported to contribute to the recognition of sentinel nodes settled in difficult anatomical areas or occult lesions (6,7). Others defended that these type of imaging systems had important limitations due to semi-flexibility, missing anatomical information, and undesirable effects such as shadowing and shine-through. Unlike the typical fixed gamma camera systems, 3D F-SPECT system is equipped with a hand-held gamma probe and an imaging camera that use a specialized software to provide a reconstructed 3D image of the sentinel node. The system provides additional in depth information and image fusion over a live video of the operation area, and allows both mobile data acquisition and real-time navigation tracking (8,9). The recent road map of intraoperative SLND was stated as real-time and 3D intraoperative imaging by two consecutive editorial comments (5,9). In the pivotal study (8), F-SPECT provided similar results when compared with SPECT/CT as a reference method. The accuracy, sensitivity, and positive predictive values were found to be 80%, 83% and 95% in the validation study, respectively. F-SPECT revealed at least one sentinel node in 87.5% of the patients. In the clinical study by Bluemel et al. (12), preoperative planar scintigraphy was used as the reference method while conventional intraoperative gamma probe was the standard alternative method. They (12) found that F-SPECT and conventional gamma probe had a detection rate of 92.3% (36/39) and 89.7% (35/39) in the evaluation on a per-patient basis and a detection rate of 92.7% (51/55) and 69.1% (38/55) in the evaluation on a per-lesion basis, respectively. There was a statistically significant difference (p<0.001) between the two methods for sentinel node detection on a per-lesion basis. Finally, although the cost of buying a F-SPECT system was reported to be approximately £92,000, the real cost-effectiveness could not be evaluated because of its potential use in numerous surgical specialties (13). The success of SLND is closely related to selected protocols and parameters. Despite all efforts to achieve a standard SLND protocol, there is still no consensus on the procedure of choice. The main limitations in creating a standard protocol are as follows: the diversity in imaging methods and in radioactive agents and injection types, rapid technological improvement, accessibility and pricing policies (1,2,4,10). 99mTc-sulfur colloid, 99mTc-nanocolloid, and 99mTc-antimony trisulfide have different particle sizes that may be a factor in SLN detection rates. However, as in our case, selection of the radiotracer is usually dependent on its local accessibility rather than other factors. Subareolar, periareolar or peritumoral injection techniques with deep (parenchymal or subcutaneous) or superficial (epidermal, subdermal or intradermal) administrations can be preferred (1,2). However, better results can be achieved with a superficial administration in subareolar or periareolar regions to visualize the axillary node, or with a deep administration in the peritumoral region to visualize the internal mammary node (14,15). In both of the major studies (8,12) the technique was based on the two-day protocol using different injection types and there was no comparison between different protocols.

## CONCLUSION

In conclusion, the concept of SLND is currently one of the standard procedures for patients with breast cancer. Application of extensive axillary dissection for the identification and removal of a SLN can cause lymphedema both in the patient’s arm and the conserved breast. The exact 3D definition is one of the new options to avoid the morbidity related to unnecessary lymphatic dissection. We tested this new method with two main protocols under stable conditions via usage of same radiotracer and injection technique and combined imaging method. In our cases, with the help of 3D F-SPECT system, we detected the SLN precisely with an error of only a few millimeters (Figure 2). In our opinion, radio-guided breast surgery may be favorable because it allows lower postoperative morbidity with shorter operation time and a quite limited dissection area. We think that 3D F-SPECT technology is a promising method that could meet expectations. The simultaneous combination of SPECT and CT or optical images in the operative area may further increase the value of the current system. However, the number of articles published so far is small, and there is no comment about the impact of this method on morbidity in the literature. Further studies in the field of radio-guided surgery and intraoperative imaging seem to be required to reveal the exact potential of these methods to gain wide clinical acceptance.

## Ethics

Informed Consent: Consent form was filled out by all participants.

Peer-review: Externally peer-reviewed.

Financial Disclosure: The authors declared that this study has received no financial support.

## References

[1] Vidal-Sicart S, Valdés Olmos R (2012). Sentinel node mapping for breast cancer: current situation. J Oncol.

[2] Boguševičius A, Čepulienė D (2013). Quality of life after sentinel lymph node biopsy versus complete axillary lymph node dissection in early breast cancer: a 3-year follow-up study. Medicina (Kaunas).

[3] Cheng G, Kurita S, Torigian DA, Alavi A (2011). Current status of sentinel lymph-node biopsy in patients with breast cancer. Eur J Nucl Med Mol Imaging.

[4] Ali J, Alireza R, Mostafa M, Naser FM, Bahram M, Ramin S (2011). Comparison between one day and two days protocols for sentinel node mapping of breast cancer patients. Hell J Nucl Med.

[5] Olmos RA, Vidal-Sicart S, Nieweg OE (2009). SPECT-CT and real-time intraoperative imaging: new tools for sentinel node localization and radioguided surgery?. Eur J Nucl Med Mol Imaging.

[6] Paredes P, Vidal-Sicart S, Zanón G, Roé N, Rubí S, Lafuente S, Pavía J, Pons F (2008). Radioguided occult lesion localisation in breast cancer using an intraoperative portable gamma camera: first results. Eur J Nucl Med Mol Imaging.

[7] Vidal-Sicart S, Paredes P, Zanón G, Pahisa J, Martinez-Román S, Caparrós X, Vilalta A, Rull R, Pons F (2010). Added value of intraoperative real-time imaging in searches for difficult-to-locate sentinel nodes. J Nucl Med.

[8] Wendler T, Herrmann K, Schnelzer A, Lasser T, Traub J, Kutter O, Ehlerding A, Scheidhauer K, Schuster T, Kiechle M, Schwaiger M, Navab N, Ziegler SI, Buck AK (2010). First demonstration of 3-D lymphatic mapping in breast cancer using freehand SPECT. Eur J Nucl Med Mol Imaging.

[9] Valdés Olmos RA, Vidal-Sicart S, Nieweg OE (2010). Technological innovation in the sentinel node procedure: towards 3-D intraoperative imaging. Eur J Nucl Med Mol Imaging.

[10] Kim T, Giuliano AE, Lyman GH (2006). Lymphatic mapping and sentinel lymph node biopsy in early-stage breast carcinoma: a metaanalysis. Cancer.

[11] Arıcan P, Peksoy I, Naldöken S, Bozkurt B (2011). The effect of the excisional biopsy in the detection of the sentinel lymph node by lymphoscintigraphy and intraoperative gamma probe in breast cancer. Mol Imaging Radionucl Ther.

[12] Bluemel C, Schnelzer A, Okur A, Ehlerding A, Paepke S, Scheidhauer K, Kiechle M (2013). Freehand SPECT for image-guided sentinel lymph node biopsy in breast cancer. Eur J Nucl Med Mol Imaging.

[13] Naji S, Tadros A, Traub J, Healy C (2011). Case report: improving the speed and accuracy of melanoma sentinel node biopsy with 3D intra-operative imaging. J Plast Reconstr Aesthet Surg.

[14] Mudun A, Sanli Y, Ozmen V, Turkmen C, Ozel S, Eroglu A, Igci A, Yavuz E, Tuzlali S, Muslumanoglu M, Cantez S (2008). Comparison of different injection sites of radionuclide for sentinel lymph node detection in breast cancer: single institution experience. Clin Nucl Med.

[15] Bauer TW, Spitz FR, Callans LS, Alavi A, Mick R, Weinstein SP, Bedrosian I, Fraker DL, Bauer TL, Czerniecki BJ (2002). Subareolar and peritumoral injection identify similar sentinel nodes for breast cancer. Ann Surg Oncol.

